# Transition Aged Individuals With Cerebral Palsy Have Larger Clinical Gains With Visual Performance Feedback During Power Training

**DOI:** 10.1016/j.arrct.2025.100463

**Published:** 2025-05-09

**Authors:** Brad Corr, Heidi Reelfs, Michael Trevarrow, Sarah Baker, Max J. Kurz

**Affiliations:** a Center for Human Performance Optimization, Boys Town National Research Hospital, Omaha, NE.; b Institute for Human Neuroscience, Boys Town National Research Hospital, Omaha, NE.; c Department of Physical Therapy, Munroe-Meyer Institute for Genetics and Rehabilitation, University of Nebraska Medical Center, Omaha, NE.; d Department of Pharmacology and Neuroscience, Creighton University School of Medicine, Omaha, NE.

**Keywords:** Gait, Lower extremity, Physical therapy, Rehabilitation

## Abstract

**Objective:**

To evaluate if providing visual feedback (VFB) on the speed of the movement during a lower extremity power training treatment protocol results in greater clinical gains in transition aged individuals with cerebral palsy (CP).

**Design:**

Nonrandomized controlled trial.

**Setting:**

Academic medical center.

**Participants:**

Twenty transition aged persons (N=20) with CP (age range, 11-24y; Gross Motor Function Classification Score [GMFCS], I-IV).

**Interventions:**

Twenty-four (8wks; 3d/wk) lower extremity power training sessions while receiving either VFB of their performance or no feedback (NFB) on their performance.

**Main Outcome Measures:**

Bilateral leg press 1-repetition maximum (1RM), bilateral leg peak power production and walking speed reserve.

**Results:**

The VFB group had greater lower extremity strength gains than the NFB group (*P*=.026). Additionally, the 1RM clinical gains were dependent on the baseline 1RM (*P*<.001). The VFB group also had greater lower extremity power production after power training (*P*=.009). The extent of the power production gains was partially dependent on the baseline power production (*P*<.001). The VFB group also had a larger walking speed reserve after the treatment (*P*=.039). However, the extent of the walking speed reserve gains was linked with an individual’s GMFCS level (*P*<.001).

**Conclusions:**

VFB during power training has the potential to results in larger clinical gains for transition aged individuals with CP. Individuals with higher GMFCS levels, lower muscular strength and muscular power at baseline might not demonstrate as large of gains after power training even when VFB is provided. Alternative treatment strategies should be considered for these cases. Nevertheless, our results convey that learning to perform fast lower extremity motor actions likely has clinically relevant benefits for transition aged individuals with CP.

Cerebral palsy (CP) remains one of the most prevalent developmental disabilities and although once simply considered an early onset static brain injury with resulting muscle weakness and spasticity, our understanding has grown to appreciate the lifelong implications.^1^ Deterioration in walking skills with age for individuals with CP is well documented in the literature, with prominent declines occurring before 35 years of age.^2^^,^^3^ This implies that when individuals are in the prime productive years of their lives they are declining in physical mobility. Mobility leads to access, access leads to participation and participation leads to social engagement. Altogether this implies that decreased mobility is closely linked to the declines in physical and mental health experienced of adults with CP. Proper management of the transition to adulthood during this critical window is important to combat the cascading effects of the mobility declines seen in this population. Unfortunately, there is a paucity of evidence-based interventions designed specifically to improve and/or maintain the mobility of individuals with CP during this transition age.

Muscle strengthening has long been used as a treatment approach for improving the mobility of those with CP, but has had mixed results.^4^ It is thought that because daily functional movements rarely require maximal effort, the ability to produce submaximal muscular forces quickly may have a greater effect on mobility. This premise has led to an interest in the clinical literature on the feasibility of power training to result in beneficial improvements in the mobility and muscular function of persons with CP.^5^, ^6^, ^7^, ^8^, ^9^, ^10^ Power training is different from strength training as it emphasizes speed of force production at submaximal thresholds, whereas strength training emphasized muscular force production at a slower speed. Power training has been reported to stimulate muscle plasticity and improve rate of force production when applied to single joint movements.^7^ Furthermore, in youth with CP, functional movement activities focused on speed, result in changes in gait and improvements in walking capacity not seen in traditional strength training.^8^^,^^10^^,^^11^ Despite the noted benefits of power training, a challenge of the treatment approach is that the fidelity of the treatment plan is unknown. This is because the therapeutic prescriptions used in the prior investigations were based on verbal instructions to perform the movement “as fast as possible” with no quantification of the movement speed. Conceptually, this approach is potentially flawed because neither the person with CP nor the treating physical therapist know if they are achieving the critical velocity threshold for meeting the key ingredients. As such, many of the prior studies may have prescribed protocols that are more similar to strength training rather than the intended high velocity associated with power training. Recognizing the potential pitfalls of the prior power training studies, we have developed system that provides the person with CP and the physical therapist with real-time feedback about the speed of the leg movement being performed on the Total Gym GTS system.^a^ The overall objective of this investigation was to evaluate if the providing feedback on the speed of the movement performed during power training results in greater clinical gains.

## Methods

### Design

The Institutional Review Board approved this investigation (IRB #082-18-FB) and the investigation was registered on clinicaltrials.gov (NCT03555708). Informed consent was acquired from all of the participants and the youth assented to participate. This study design was a nonrandomized controlled trial with parallel treatment groups that consisted of a visual feedback (VFB) group that received information on the velocity of their performance or a no visual feedback (NFB) group. Participants were assigned to the respective treatment groups with the intention to balance the Gross Motor Function Classification Score [GMFCS] levels and age of the respective groups by the principal investigator (ie, lead author). Bilateral leg extension 1-repetition max (1RM), bilateral leg peak extension power, and the walking speed reserve were the primary outcome variables. The respective outcome measures were collected 1-week before starting the treatment and within 1-week after completing the 8-weeks of the intervention.

### Participants

Using the smallest effect size (d=1.69) seen for the change in lower extremity power and 1-repetition maximum for the participants with CP that completed in a prior lower extremity power training protocol,^5^ 8 participants would provide >80% power to detect a similar difference at a 0.01 alpha level. Participants for this investigation were recruited by referral from outpatient and school-based clinical service providers at University of Nebraska Medical Center’s Munroe-Meyer Institute for Genetics and Rehabilitation in Omaha, Nebraska. Participants were excluded if they had insufficient cognition to use the feedback and/or follow the physical therapist instructions, orthopedic surgery within the last 6 months and/or botulinum injections within the last 6 months.

The sample of participants included 20 transition age individuals with CP that had either spastic hemi or diplegic type presentations. None of the participants were in an exercise program before enrollment and were not undergoing concurrent physical therapy. Nine of the participants were assigned to the VFB group (age, 16.0±1.3y; GMFCS, I-IV; 4 women) and 11 were assigned to the NFB group (age, 17.5±1.3y; GMFCS, I-IV; 7 women). The number of participants in the respective treatment groups was unbalanced because of the COVID-19 pandemic halting our recruitment and overall study progress. Nonetheless, there were no significant differences between the GMFCS levels (*P*=.33) and age (*P*=.51) of the respective groups. Further details on the participants are shown in table 1.

**Table 1:** Demographics of participants included in this investigation and group results.

Characteristic	No Visual Feedback	Visual Feedback	*P* Value
Age (y)	17.5 (1.3)	16.0 (1.3)	.51
Sex			
Female	7	4	
Male	4	5	
Race			
African American	4	0	
White	7	8	
NA		1	
GMFCS level			.33
I	4	6	
II	3	1	
III	2	1	
IV	2	1	
1RM (N)			
Pre	530.25 (107.5)	828.4 (121.0)	
Post	732.54 (125.2)	1402.92 (223.2)	**.026**
Peak muscle power production (W)			
Pre	353.96 (68.7)	536.70 (75.9)	
Post	382.74 (66.6)	777.54 (117.4)	**.009**
Walking speed reserve (m/s)			
Pre	0.35 (.011)	0.54 (0.08)	
Post	0.31 (0.07)	0.77 (0.15)	**.039**

Data presented as mean and standard error of the mean.

Abbreviation: NA, not applicable.

Bolded p values indicate p<0.05.

### Power training intervention

The 2 treatment groups performed 24 sessions of power training (3d/wk × 8wk) consisting of unilateral and bilateral leg presses on a Total Gym GTS system. The treatment guidelines followed the American College of Sports Medicine’s power training position statement that recommends the training loads should be between 30% and 60% of an individual’s 1-repetition maximum (1RM) and the concentric portion of the movement should be performed as fast-as-possible. Power is the product of the load lifted and the velocity of the movement. The treatment plan was directed at scaling the velocity of the movement as opposed to the load. However, when the participant appeared to achieve a threshold velocity for the given load, the physical therapist increased the load lifted for the subsequent set. The progression of the load and velocity parameters to achieve a participant’s optimal challenge point was done at the physical therapist visual inspection of the movement performance for the NFB group, whereas the speed feedback system was used for the progression used by the physical therapist treating the VFB group. Separate physical therapists treated the respective groups.

**Fig 1:**
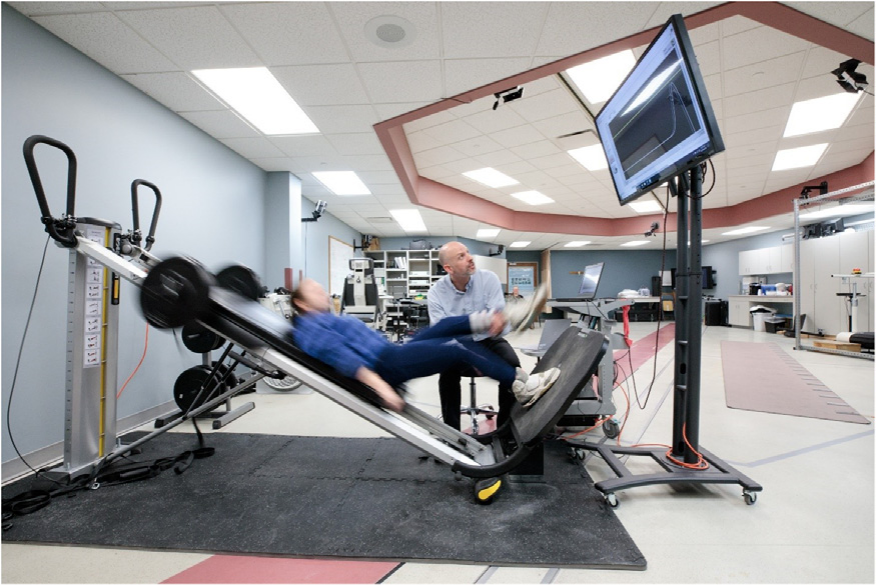
Depiction of a participant in the visual feedback group undergoing power training on the Total Gym GTS system. As shown, the physical therapist and participant viewed a monitor that provided information about the speed of the leg press and the target speed the therapist prescribed for the set.

We retrofitted the Total Gym GTS with a cable actuated position sensor^b^ that was connected to a data acquisition board.^c^ A custom LabView program collected the sensor output and differentiated the cable displacement to quantify the speed of the leg press performance in real-time. The participants in the VFB group and the treating therapist receive real-time VFB of their movement speed on a large monitor that was positioned in front of the Total Gym GTS system (fig 1). Secondarily, the custom software allowed for the physical therapist to set a target speed that the participant was instructed to exceed. The target speed was based on the therapist’s assessment of the patient’s optimal challenge point, with the goal of achieving and the exceeding lower extremity speeds for sit-to-stand movements (0.3-0.4m/s) and stair climbing (eg, 0.49-0.56m/s) that are reported in the biomechanical literature.^12^, ^13^, ^14^, ^15^

The target speed and rep by rep tracing of the speed was shown to the VFB group on a monitor placed in front of the Total Gym GTS, whereas no feedback (NFB) on the movement performance was shown to the NFB group. Participants performed 6 sets of 5-repetitions at 30%-60% of 1RM for each leg separately. After the unilateral leg presses, 6 sets of 5-repetitions of bilateral leg presses were performed at the predetermined percentage of 1RM. Instructions for the concentric phase of the leg press were to push as fast-as-possible with the eccentric portion performed slow and controlled over approximately 1 to 2 seconds. Weights were added to a weight bar to increase resistance, and the training load started at 30% of 1RM in session 1 with the goal of progressing the load lifted to 60% by the final week of the 8-weeks of treatment.

### Muscular function test

The bilateral leg press 1RM assessment was performed on the Total Gym GTS with the sled positioned at 45°. Participants were positioned with the hips and knees at 90° and instructed to perform a leg press by extending the knees through full range of motion. The 1RM was the final weight lifted in a single repetition.

A separate test was used to assess the participant’s lower extremity power while performing a bilateral leg press on the Total Gym GTS system. The output of the linear transducer cable sensor that was integrated into the Total Gym GTS system was used to determine the movement velocity of the sled during the power assessment. Power was calculated as the product of the load lifted multiplied by the velocity of the leg press, while taking into consideration of the 45° angle of the Total Gym GTS system. Maximum power was determined from 5 consecutive repetitions that were performed at 40% of the participant’s baseline 1RM.

### Walking speed reserve

Three trials were completed at the participant’s preferred and fast-as-possible walking speeds over a digital mat^d^ based under the guidelines of the 10-m walk test.^16^ The participant’s used any mobility aides they would typically use in the community or in the home environment (ie, forearm crutches, wheeled walker) for the respective tests. The fastest speeds recorded across the 3 trials for the preferred and fast-as-possible assessments were used to calculate the participant’s walking speed reserve. The walking speed reserve represented the participant’s capacity for altering their walking speed. The walking speed reserve at the baseline was calculated as the difference between the baseline fast-as-possible walking speed and the baseline preferred walking speed. Conversely, the walking speed reserve measured after physical therapy was calculated as the difference between the posttherapy fast-as-possible walking speed and the baseline preferred walking speed. Essentially, this normalized the assessment of the participant’s ability to change their walking speed at the respective timepoints to the baseline preferred walking speed.

### Statistical analysis

Separate univariate general linear models with the baseline assessment and GMFCS level as covariates were used to determine if there were differences between the VFB and NFB groups at the post-treatment timepoint for the respective outcome variables. Including the respective baseline outcome variable as covariates in our final statistical analysis accounted for possible differences in groups before undergoing treatment and reduced the potential effects of allocation bias. Final statistical analysis was not performed by a blinded assessor.

## Results

**Fig 2:**
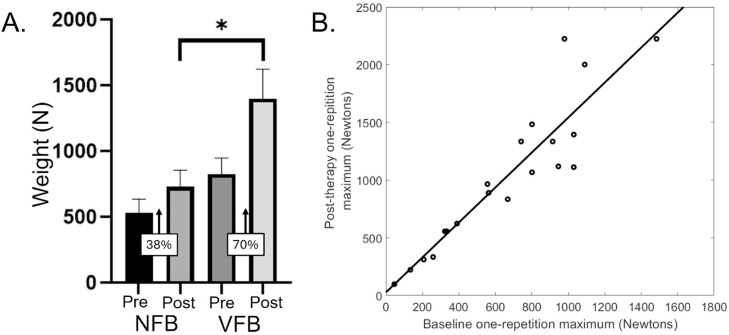
(A) One-repetition maximum (1RM) on the Total Gym GTS system for a bilateral leg press at the pre (ie, baseline) and postassessment timepoints for the no feedback (NFB) group and visual feedback (VFB) group. As shown, the VFB group had substantially greater improvement in their 1RM after the physical therapy. When controlling for GMFCS level and the 1RM at baseline, the VFB group had a larger 1RM for the posttherapy assessment than the NFB group. *P=.026. Data presented as the mean ± standard error of the mean. (B) Scatter plot of the relationship between the baseline 1RM and the posttherapy 1RM. As depicted, an individual with a lower 1RM at baseline is likely to have lower clinical gains at the posttherapy 1RM.

The group results at the pre-therapy and post-therapy timepoints are shown in table 1. The participants in the NFB group had a 38% improvement in their 1RM, whereas the VFB group had a 70% increase in their 1RM after the power training (fig 2A). Our statistical analysis indicated that the VFB group had greater lower extremity strength than the NFB group after the treatment (F=6.02; *P*=.026). Additionally, the baseline 1RM covariate was significant (F=24.65; *P*<.001), whereas the GMFCS covariate was not (F=1.26; *P*=.278). Scatter plot of the relationship between the baseline 1RM and post-therapy 1RM suggests that an individual with a lower 1RM at baseline is likely to have lower clinical gains in their muscular strength at the post-therapy timepoint (fig 2B).

**Fig 3:**
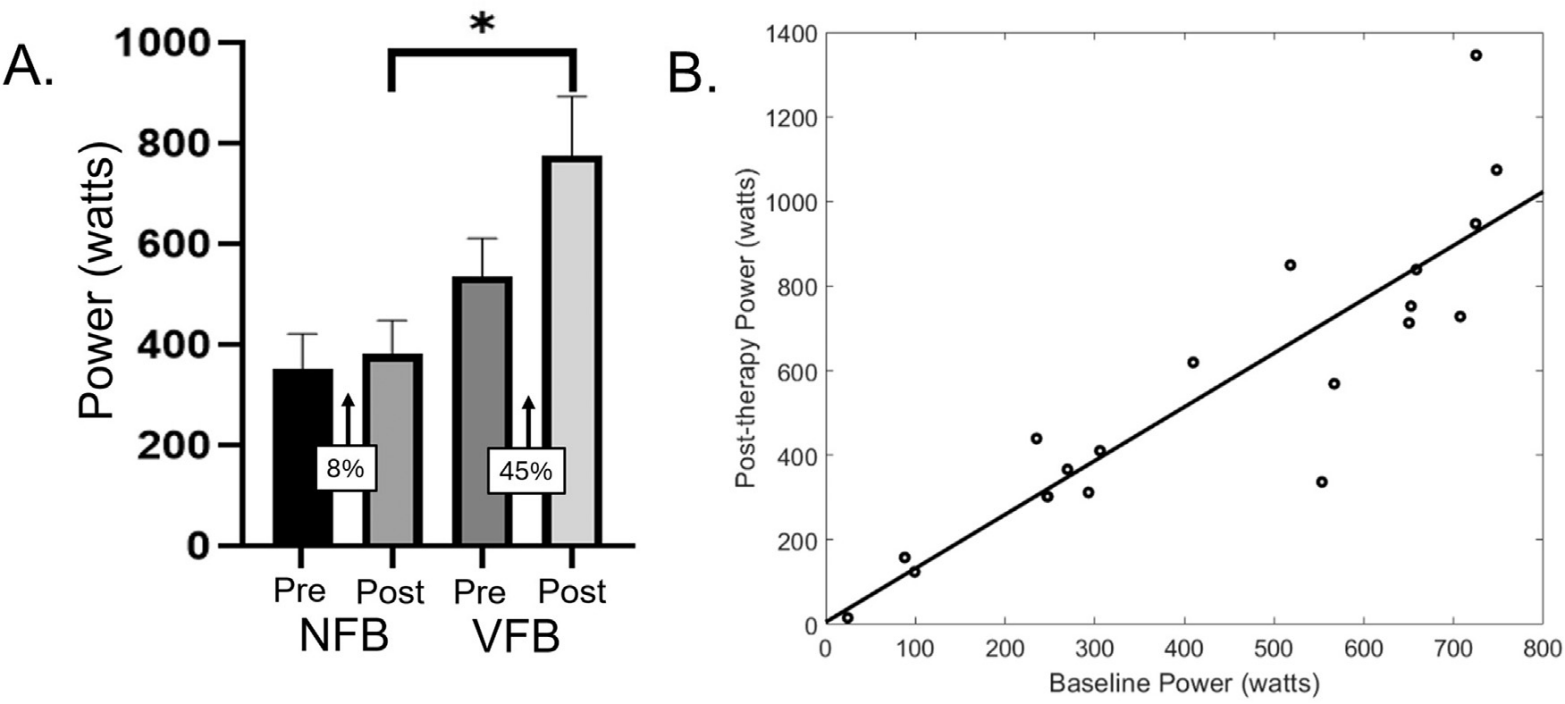
(A) Bilateral leg press power on the Total Gym GTS system at the pre (ie, baseline) and post-assessment timepoints for the no feedback (NFB) group and visual feedback (VFB) group. As shown, the VFB group had substantially greater improvement in their leg power production after the physical therapy. When controlling for GMFCS level and the leg power production at baseline, the VFB group had a larger power production for the post-therapy assessment than the NFB group. *P=.009. Data presented as the mean ± standard error of the mean. (B) Scatter plot of the baseline bilateral leg power production and the post-therapy bilateral leg power production on the Total Gym GTS system. As shown, an individual that is capable of generating less power with their lower extremity at baseline is likely to show lower clinical gains in their power production after treatment.

The VFB group also had a 45% improvement in their lower extremity power production, whereas the NFB group remarkably only had an 8% change (fig 3A). The statistical analysis indicated that the VFB had greater lower extremity power production when compared with the NFB group after the treatment (F=8.75; *P*=.009). The baseline leg power production covariate was significant (F=20.60; *P*<.001); yet the GMFCS covariate was not (F=1.13; *P*=.302). The scatter plot of the relationship between the baseline and post-therapy leg power production suggested that an individual capable of generating less power with their lower extremity at baseline was likely to show lower clinical gains in their power production after treatment (fig 3B).

**Fig 4:**
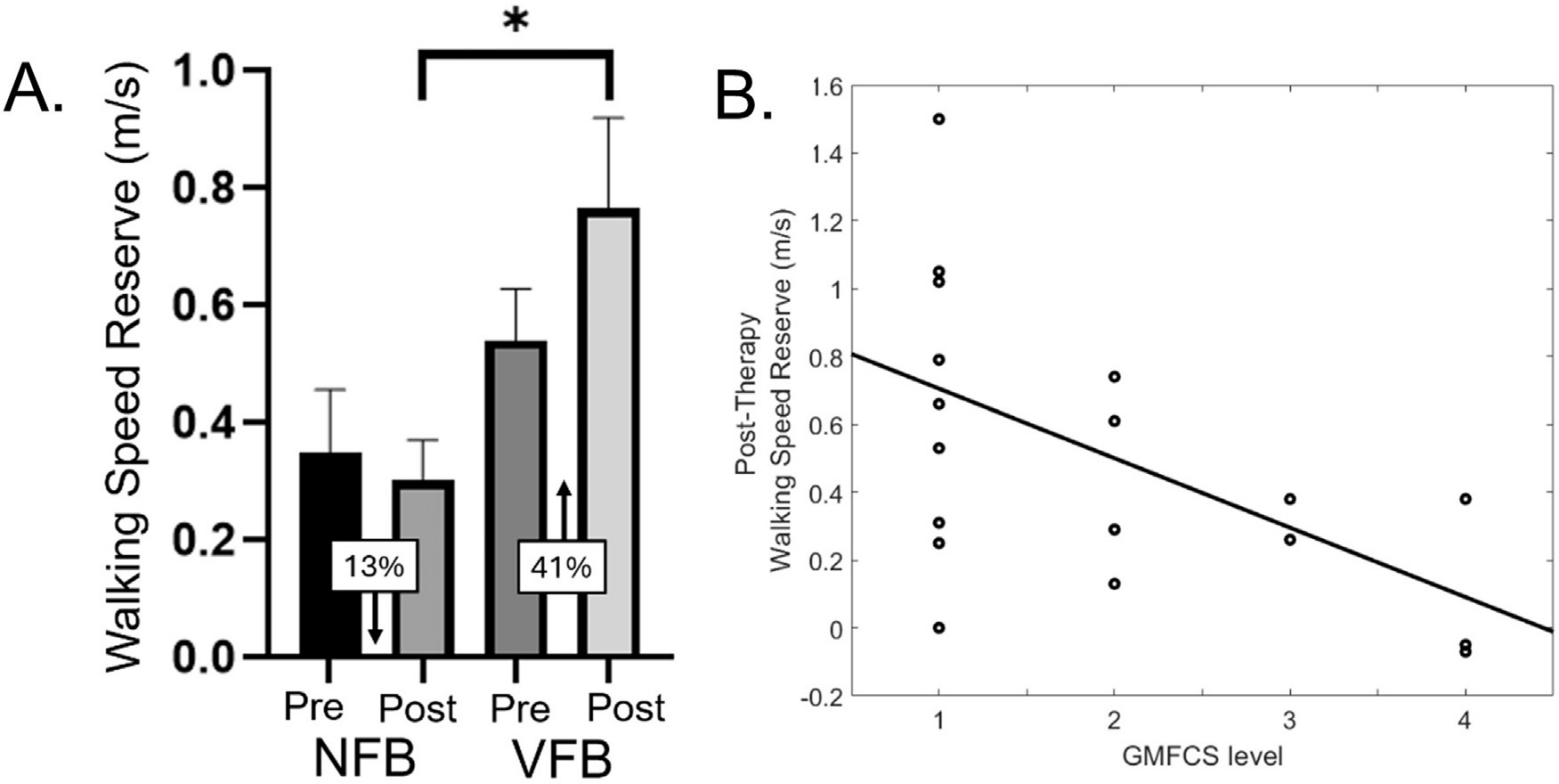
(A) Walking speed reserve for the pre (ie, baseline) and post-assessment timepoints for the no feedback (NFB) group and visual feedback (VFB) group. As shown, the VFB group had substantially greater improvement in their walking speed reserve after the physical therapy. When controlling for GMFCS level and the leg power production at baseline, the VFB group had a larger improvement in their walking speed reserve for the post-therapy assessment than the NFB group. *P=.039. Data presented as the mean ± standard error of the mean. (B) Scatter plot of the relationship between the participant’s GMFCS level and post-therapy walking speed reserve. As shown, an individual with a higher GMFCS level is likely to display less changes in their walking speed reserve after treatment.

Lastly, the VFB group had a noteworthy 41% improvement in their walking speed reserve, whereas the NFB group displayed a 13% reduction (fig 4A). The statistical analysis indicated that the VFB group had a greater capacity for changing their gait speed than the NFB group after the treatment (F=5.10; *P*=.039). The baseline walking speed reserve covariate was not significant (F=0.25; *P*=.623). However, the GMFCS covariate was significant (F=6.71; *P*=.02). The scatter plot of the relationship between the GMFCS level and post-therapy walking speed reserve indicated that those with higher GMFCS levels were likely to display less changes in their walking speed reserve after treatment (fig 4B).

## Discussion

The participants in the VFB group and NFB group had a larger 1RM after undergoing the power training protocol, implying that the power training has the potential to improve the overall strength of transition aged individuals with CP. These results are aligned with the prior literature that has performed power training with youth with CP.^5^, ^6^, ^7^^,^^16^ However, our results show that the magnitude of the improvement was substantially larger for the VFB group when compared with the NFB group (ie, 70% vs 35%), which conveys that the knowledge of the speed of the movement performance played a potent role in the strength gains. We suggest that the feedback provided positive reinforcement as the participants learned how to successfully recruit the motor units for generating forceful concentric muscular contractions. Secondarily, the VFB likely ensured that the participants were receiving the intended treatment stimulus that targeted the type II motor units that play a prominent role in the generation of fast and forceful muscular contractions. Such a neurophysiological stimulus would be remarkably beneficial as prior research has shown that adults with CP lack the ability to excite the type II motor units.^17^

The participants in the VFB group also had larger clinical gains in their lower extremity power production when compared with the NFB group (45% vs 8%). The lack of a change in the power production of the NFB group implies that they might have received a treatment protocol that was more similar to strength training than the intended high velocity treatment protocol. As such, the group differences in the clinical gains reflect differences in the type of motor action that was practiced. Essentially, velocity-based power training is a novel concept for both the provider and participant, and without knowledge of the movement speed being performed, both the provider and participant may not be adequately equipped to shift from the traditional strength training approach to the intended velocity-based power training. This shift might be more difficult for individuals with CP, as it has been documented that many have uncharacteristic sensory processing at the spinal cord and cortical level.^6^^,^^18^, ^19^, ^20^, ^21^ Principally, the VFB provided during power training may help transition aged individuals with CP overcome these deficits and improve their ability to couple the relationship between their motor actions and the sensory feedback returned.

Our results also suggest that individuals with lower muscular strength and power production in their baseline assessments are less likely to display as large of clinical gains after undergoing power training. For one, these results imply that those with low strength and/or power production during their initial clinical assessment may not be good candidates for power training and alternative treatment approaches should be considered. However, the reason that these individuals do not respond as well to power training is not clear. One would tend to speculate that those with higher GMFCS levels might be the nonresponders. However, our results convey that GMFCS level does not appear to play a role in the extent of the muscular function gains. The neurophysiological reason for the lack of change in those classified as being nonresponders remains as a primary gordian knot in the physical therapy field. Identifying the neurophysiological barriers of these participants will derive new individualized treatment strategies that will enable all transition aged individuals with CP to have a breakthrough in their muscular function and mobility.

Remarkably, the participants in the VFB group also had a 41% improvement in their walking speed reserve, whereas the NFB group had a slight reduction. These results imply that practicing fast leg presses can be translated to the ability to improve the range of walking speeds that transition aged individuals with CP are capable of producing. It is postulated that the ability to produce muscle power quickly is more essential than maximal strength in functional mobility.^22^^,^^23^ A better walking speed reserve demonstrates an individual’s ability to call upon the required resources when needed to navigate environmental demands. For individuals who may never have experienced autonomous opportunities to move as fast as possible, practicing fast movements may be a novel concept and challenge their perceptions of how fast they can move. Providing real-time feedback on the speed of the leg movements during training as well as progressing target speed may afford an individual critical knowledge of results to couple new motor perception and action plans, which contribute to changes seen beyond the practiced task alone and extending into changes in functional mobility. The lack of change in the NFB group was still perplexing, and gait analysis with electromyography is suggested to provide better insight on why the treatment was less beneficial.

Our results also conveyed that individuals with higher GMFCS levels are less likely to show a larger walking speed reserve after power training than those with lower GMFCS levels. This might suggest that transition aged individuals with CP with higher GMFCS levels have less capacity for changing their walking speed reserve. Furthermore, this provides some insight on why some individuals with CP might appear to be nonresponders on gait outcome measures after undergoing power training and highlights the need for alternative treatment approaches for those with higher GMFCS levels if the goal is to improve an individual’s walking speed reserve. For example, the employment of a short-burst interval gait training treatment strategy might result in larger clinical gains in the walking speed reserve of those with higher GMFCS level because the task practiced matches the intended goal.^24^

## Conclusions

Monitoring the speed of the movement during a lower extremity power training protocol has the potential to result in larger clinical gains in the muscular function and mobility of transition aged individuals with CP. However, individuals with lower muscular function and higher GMFCS levels might not demonstrate as large of gains after power training. Despite these considerations, our results convey that learning to perform fast lower extremity motor actions likely has clinically relevant benefits for transition aged individuals with CP.

## Suppliers

a. Total Gym GTS; Total Gym Fitness.
b. SGD-120-3; TE Connectivity.
c. LabView; National Instruments.
d. GAITRite; CIR Systems.

## Disclosure

The investigators have no financial or nonfinancial disclosures to make in relation to this project.
